# Supplementary material for: MetaRibo-Seq measures translation in microbiomes
Source: Nat Commun. 2020 Jun 29;11:3268. doi: 10.1038/s41467-020-17081-z (PMC7324362; doi:10.1038/s41467-020-17081-z)
Supplement: Supplementary file 10 — Supplementary Data 7 [file 41467_2020_17081_MOESM10_ESM.zip › File2/Confidence_High_Taxonomy/367795_out.krona.html]

Javascript must be enabled to view this page.

members
magnitude
magnitudeUnassigned
count
unassigned
taxon
rank

367795\_out

48

48
2
superkingdom

1239
phylum
48

48
186801
class

48
order
186802

family
216572
47

47
459786
genus

species

SRS049896\_contig\_number\_15650SRS057480\_contig\_number\_contig-100\_28048.28049SRS104400\_contig\_number\_44361SRS892882\_contig\_number\_11030
1897012
4

5
1262911

SRS018541\_contig\_number\_contig-100\_55.97096SRS019968\_contig\_number\_contig-100\_1447.105441SRS105153\_contig\_number\_29480SRS147425\_contig\_number\_8445SRS893279\_contig\_number\_16161
species

38
species
1945593

SRS014459\_contig\_number\_32548SRS014613\_contig\_number\_5552SRS014948\_contig\_number\_23796SRS015065\_contig\_number\_contig-100\_10602.52098SRS015782\_contig\_number\_11265SRS043768\_contig\_number\_30338SRS046717\_contig\_number\_10741SRS049773\_contig\_number\_13807SRS049959\_contig\_number\_12670SRS050752\_contig\_number\_contig-100\_7927.140398SRS054905\_contig\_number\_24433SRS055017\_contig\_number\_13611SRS064276\_contig\_number\_6677SRS077294\_contig\_number\_13549SRS077392\_contig\_number\_11306SRS100907\_contig\_number\_contig-100\_3692.3693SRS103987\_contig\_number\_18981SRS1041145\_contig\_number\_26043SRS105082\_contig\_number\_17037SRS1055049\_contig\_number\_14388SRS142599\_contig\_number\_24421SRS143342\_contig\_number\_19767SRS143876\_contig\_number\_36912SRS144183\_contig\_number\_28632SRS144506\_contig\_number\_19515SRS144603\_contig\_number\_contig-100\_2499.46145SRS146813\_contig\_number\_6818SRS146888\_contig\_number\_3594SRS147271\_contig\_number\_22648SRS147977\_contig\_number\_10522SRS148319\_contig\_number\_16405SRS148424\_contig\_number\_28309SRS892882\_contig\_number\_contig-100\_6304.89458SRS893231\_contig\_number\_contig-100\_1077.40288SRS893252\_contig\_number\_contig-100\_2848.111874SRS893295\_contig\_number\_9805SRS893300\_contig\_number\_contig-100\_2947.39412SRS893373\_contig\_number\_3211

1
family
541000

1
genus
216851

species
853

SRS011302\_contig\_number\_16882
1
